# Negotiating access to community-based participatory research

**DOI:** 10.1007/s00127-023-02583-0

**Published:** 2023-11-15

**Authors:** Annahita Ehsan

**Affiliations:** ESRC Centre for Society and Mental Health, King’s College London, London, UK

**Keywords:** Community-based participatory research, Social capital, Access, Mixed methods, Reflexivity

## Abstract

**Purpose:**

Community-based participatory research (CBPR) that improves social capital can be a powerful tool for promoting mental health and well-being. This work explores what gaining, maintaining, and losing access to this type of CBPR looks like from a reflexive research perspective.

**Method:**

I describe and reflect on my experiences conducting a mixed-methods study of an existing CBPR to increase social capital in Switzerland. I draw on ethnographic observations, field notes, and reflexive memos collected during fieldwork between 2016 and 2020.

**Results:**

I negotiated access to the CBPR across three levels: (1) formal organizational with intervention leaders, (2) implementational with facilitators, and (3) the community/group level with participants. Intervention leaders let me conduct research if they benefitted from my work in a timely and reinforcing way, facilitators granted access if I made myself helpful and supported their work, and community members accepted me if I participated in their community meaningfully. I lost access when my findings posed a potential risk to the intervention funding.

**Conclusion:**

I highlight how access is a fluid and complex process that can change throughout CBPR. I show the importance of reflexive analysis to understand how access is negotiated in diverse settings, what sources of social capital are needed to engage in these negotiations, and how positionality and power play a role in this process.

## Introduction

Community-based participatory research (CBPR) refers to a variety of research practices (e.g., Action Research, Participatory Action Research, collaborative inquiry, and others) that rely on collaborative and equitable partnerships in all phases of the research process [1, 2]. In health equity research, CBPR has emerged as an alternative research paradigm that integrates social action to improve outcomes within a community [3]. A central aspect of using CBPR as a method is establishing a reciprocal relationship between researchers and the community for transformative purposes [4]. CBPR initiatives are often used to empower or share power with equity-deserving communities. It is a process that can create bridges between communities and researchers through the process of sharing knowledge and experiences, ultimately benefiting everyone involved [5].

Engaging in CBPR can blur lines and relationships between researchers and communities, which can have implications for research ethics and appropriate safeguards [6]. For example, researchers strive to publish results, but there are ethical implications of disseminating results when it could be damaging to the community [1]. Difficulties balancing community values with individual ones, as well as negotiating power dynamics and relationships can also become ethical concerns [7]. This is particularly true when there are two spheres dividing researchers and communities, with inherent power imbalances that prevent true allyship [4]. There are many accounts of when CBPR ‘goes wrong’, or when these relationship boundaries and corresponding power dynamics become blurred [8]. Strong and trusting social relationships are seen as paramount to sharing power and conducting good CBPR [9], and reflecting on these relationships can illustrate some of the power, positionality, and ethical dilemmas that may arise [10, 11].

CBPR methods acknowledge that trusting relationships and strong partnerships are the building blocks required to conduct good CBPR [9]. Social capital is an umbrella term referring to the variety of social resources that are embedded within social relationships [12], though there are multiple definitions, applications, and understandings of the term [13]. What is less explored is that the process of engaging in CBPR often strives to increase forms of social capital in and of itself. For example, the CBPR method may be used to increase social cohesion in a community or create social bridges between communities. CBPR may also build social capital to achieve a goal, such as strengthening social ties to improve a health outcome. This begs the question: If forms of social capital (e.g., trust) are required to conduct CBPR to build social capital, are there not inherent gaps in CBPR processes?

This paper aims to highlight challenges in access to CBPR from a social capital perspective. It does so by drawing on fieldwork that aimed to study a CBPR designed to increase social capital. During this process, I began to challenge the idea that all communities are inherently poised to conduct practices based on trust and reciprocity. I observed the “dark side” of social capital, or when social relationships that are beneficial to some are detrimental to others [14, 15], and when individuals do not have enough social capital to get involved. At the same time, I required a certain amount of social capital to study the CBPR, and experienced prejudice based on my positionality as a young woman of colour who was an outsider in the community. These experiences enabled me to reflect on how access to CBPR is negotiated using various forms of social capital and contribute to the literature by showing how key assumptions in CBPR should be challenged.

### Context

This manuscript is based on research where I collaborated with a CBPR intervention in one community in Switzerland. It was part of a broader series of CBPR interventions implemented in 2002, with the initial goal of integrating older adults in their neighbourhoods, developing solidarity and connectedness with one another, and improving their overall quality of life. The series of interventions was created in response to an absence of participatory programs that prioritized older adults’ social connectedness and well-being.

Municipalities could apply to have the program in a neighbourhood or village. They could receive the intervention if they agreed to provide funding and other needed resources (e.g., provide an accessible community space). Once funded, the intervention would last between three to five years. The intervention was primarily delivered through facilitators who applied the intervention method to each community: They organized gatherings, conducted interviews with residents, facilitated focus groups and discussions, and liaised with organizations to recruit residents who would participate in the first phase of the intervention (herein referred to as participants). Over time, participants would gain the skills to take over facilitation and leadership, leading to a sustainable intervention.

## Methods

I was collaborating with the CBPR intervention to explore whether it was associated with an increase in social capital and mental health. Social capital is a known determinant of mental health [13], and I wanted to understand whether existing CBPR initiatives that improve social capital could improve mental health, informing health policy and planning [16]. To do so, I added a monitoring and evaluation component to the existing CBPR. I chose to use an embedded mixed-methods design, combining a pre–post survey with ethnographic observations of the intervention between quantitative data collection points [17]. The broader research project was done in the context of my doctoral research, which was funded by the University of Lausanne. I had ethical approval from the Swiss Ethics Board in the Canton of Vaud.

This manuscript focuses on my experiences negotiating access to the above research to highlight challenges that can arise when working with CBPR. A key challenge was adding a mixed-methods research component to a pre-existing intervention. Even though various guidelines exist for conducting CBPR [18], I was studying an existing CBPR with methods developed by the organization itself. Therefore, I had the added difficulty of being positioned as an outside research collaborator who had to access an ongoing CBPR guided by a different organization, while also recognizing that I was ‘studying’ the CBPR [19, 20]. I was also juxtaposing (1) ethnography where I was both observing the intervention and directly interacting with participants, and (2) a quantitative pre/post-survey where I was further removed from the scene, but still analysed participant data that informed my perception of the field.

I conducted over 82 h of observations and informal interviews on one site. Approximately 40–50 individuals attended participant groups, but I was also able to observe community forums with broader audiences (approximately 250 residents) and a separate group with ten representatives from local associations and municipal authorities who discussed the intervention's progress. I combined my fieldnotes with minutes from all group meetings, as well as the qualitative *additional comments* section from a community survey I had conducted, providing additional insight from individuals who did not participate in the intervention (*n* = 102 in Wave 1, and *n* = 72 in Wave 2, with 27 comments being provided by the same respondents).

I draw from data and reflexive memos collected between 2016 and 2020 to illustrate access as an ongoing process that is constantly negotiated. I describe how conducting mixed-methods research became a resource in these negotiations and outline challenges I faced while negotiating access with various informants, as well as how access was ultimately rescinded. Important themes throughout these negotiations include the power dynamics between partners who held different forms of social capital, with emphasis on intersectional positionality and my position as an ‘outsider’ [21, 22]. These experiences led me to question the process of developing social capital for individuals with different identities, as well as some limitations of CBPR.

## Results

### Processes of negotiating and maintaining access to the intervention

#### Negotiating access at the organizational level: The intervention

My supervisor arranged for me to work with the intervention through his social networks. He arranged for one of the organization’s executives to interview me, and they agreed to let me study one of their intervention sites for my Ph.D. and publish the findings. In exchange, I would quantify some of their work and provide an additional perspective to the CBPR. I implicitly understood that if the research had positive findings, it could be used as external validation for what they were doing.

Soon after stepping on board, a colleague told me that they had overheard facilitators discussing the university’s involvement in their project as a form of legitimacy. A few months later, one of the executives presented some of my findings to other organizations at a conference. They also asked me for data and analyses for progress reports. This was the first negotiation: *I could conduct my Ph.D. research with the intervention if the organization benefitted from my work in a timely and reinforcing way.*

#### Negotiating access at the implementational level: Facilitators

Even though I had organizational approval to study the intervention, I later learned that executives had not asked facilitators if they were comfortable with my research. This resulted in some friction, as how I accessed the intervention influenced my ability to build mutually trusting relationships. Specifically, I faced some hostility from one facilitator, who did not want me around. They did not communicate necessary information with me and ignored my messages, leading me to miss the first two intervention sessions. I contacted a supervisor who permitted me to show up at the next session, but this was one of the worst things I could have done to earn the facilitator's trust: The way I accessed the field could have resulted in a ‘failure’ to conduct good field research [23, 24]. I spent months trying to establish a better relationship with this facilitator, who was ultimately a gatekeeper to others.

I understood that having a researcher present was uncomfortable for the facilitator. They were explicit about where my role began and ended: they were the experts, and I was a *student* researcher. I explained that I was not there to monitor or evaluate their job performance, but to watch the processes and group dynamics that took place within the intervention and its participants. I made a conscious effort not to overstep the boundaries they had set for me, and to help where I could. For example, helping set up and clean the room before and after meetings was one way to show the facilitators that I respected them and their time, while simultaneously allowing me to talk to them without participants present. The facilitator who mistrusted me made many comments about my eagerness to help and often joked about how ‘lucky’ they were to have someone in academia bring them a coffee or put away chairs. Over time, the tone of the comments became increasingly more positive, and I started to feel a small shift. The facilitator eventually left their position, and my interactions with the other facilitators became more relaxed.

The other facilitators started asking for my opinions, and we often built on each other’s observations to make sense of what had happened during the intervention. I learned that they were tired and overworked because they had been assigned a larger geographic space than they were used to, without the additional resources or personnel that they needed. I had experience working with communities and leading activities, so I could sometimes assist. While they had initially been very stern about my role as a student researcher, they started to share some responsibility and give me more ‘hands-on’ experience. I facilitated some focus groups for them and typed minutes when asked. This made me feel like a part of the team, but it also increased my ability to qualitatively study the intervention. This was the second negotiation*: I could observe the intervention more easily if I made myself somewhat useful to the facilitators.*

Even though observing the intervention was a struggle, the quantitative portion of my research was more appreciated. The facilitators were keen to know survey findings, even if they felt that collecting this information might be ‘too much’ and could initially overburden some community members. I discussed which questions to include with the facilitators, and I conducted analyses that helped respond to their questions and draw associations between variables. The facilitators told me that the larger dataset helped validate what they were hearing in interviews, but it also provided information from people they were unable to interview. This was the third negotiation: *I could distribute a survey if I would also conduct analyses that supported the facilitators.*

#### Negotiating at the community level: Participants

The facilitators granted me access to the field by getting the municipality’s permission and facilitating my access to participants. This was essential, as I quickly learned that I would have otherwise been unwelcome in the community. Ironically, this process led me to question the principles of CBPR as having an innate ‘ethic of inclusion’ [25], and the sources of power researchers typically hold. I felt that my intersectional positionality played an important role throughout my fieldwork: I was born in Canada to parents who immigrated from Iran, and I am darker-skinned. While I was able to teach and work in French, French is my third language and I speak it with an accent. I was an outsider and the only thing I could change to ‘fit in’ was the way I dressed and presented myself.

It became clear that there were some feelings of xenophobia in the community. The open-ended areas on the first questionnaire had responses highlighting how the town was being “*invaded by Americans, Russians, and Chinese people”*. Others highlighted that the best thing to do for the town would be to get rid of outsiders. At one of the first meetings I attended, someone vocally stated that they should *“assimilate”* the outsiders. A municipal leader refused to let us distribute questionnaires in English because people in the town were supposed to speak French. In short, various aspects of my research quickly revealed that there was considerable dislike of *“expats”* (their term) and some community members were actively excluding non-Francophones from taking part. My identity and positionality as someone they already disliked made engaging with this community particularly difficult. I was unsure what to do, especially because CBPR guidelines often emphasize the researcher as having multiple dimensions of power (e.g., Whiteness) and working with marginalized communities.

While Switzerland is not known for being particularly diverse, the disdain for ‘expats’ seemed particular to this town. I had visited other intervention sites in different towns to prepare for my fieldwork, where participants had been warmer and vocally curious about my origins and why I was there. Even if there were interactions that made me uncomfortable at times (e.g., insisting I go on a date with a man at least twice my age because they thought we had the same ethnic background), people were willing to interact with me. In this intervention, I felt that some residents were actively put off by me and avoided asking me any questions about where I was from or why I was there. It was only after a couple of months that participants felt comfortable enough to ask where I was really from, why I didn’t have a Québécois accent, or why I had darker skin if I was from Canada. Of course, some participants never warmed up to me, and one woman even refused to shake my hand or acknowledge me when I introduced myself.

Even though my identity made things harder to navigate, I held a form of power through my dual positionality as a student and a researcher. I had already negotiated access with the intervention facilitators and had been introduced as someone who would be following the project for a while. Being a researcher gave me some legitimacy in participants’ eyes, but being a student was equally, if not more, valuable. I think that my willingness to learn about their culture and the opportunity for them to share knowledge with me encouraged some participants to be more helpful than they might have been otherwise. Some even took on a protective, almost grandparental role by the end of my fieldwork. This dual status enabled me to overcome some of the barriers that my intersecting identities created.

Gaining the community's trust was a slow process, but I was able to build positive relationships over time. Many residents had lived in other countries, seemed amused by my interest in their small town, and were happy to share their experiences and culture with me. I think that I gained trust by regularly attending meetings and by showing that I was willing to improve my French, learn their customs, and contribute to their community. One facilitator noted that residents started warming up to me after I brought Nanaimo bars to share with everyone. I also think they enjoyed more subtle things, like when I showed a preference for a regional white wine and when I laughed at local jokes.

Despite the smaller group dynamics, I felt I earned the most trust when I presented some of my quantitative findings at a community forum. Participants had asked me to share my findings with them numerous times, and I felt they warmed up to me once they saw I was true to my word of sharing information back into their community. I started to feel much more comfortable and had more friendly interactions with participants. For example, some started to give me three pecks on the cheek for both hello and goodbye, which was symbolic of acceptance.

Some participants became invested in my student trajectory and tried to help me on different occasions. For example, one participant offered to edit my French texts multiple times, and another, a retired anthropologist, often checked in to see how I was finding my fieldwork. Once I had left a book on site, and when I returned to retrieve it, the room was locked. As I was trying to convince the concierge to let me in, one of the participants pulled up on a motorbike and asked me how he could help and if I needed a ride to the train station. It was moments like these where I felt truly accepted by some participants. This brings me to my fourth and final negotiation:* Community members were willing to accept me with open arms if I participated in their community meaningfully.*

### Losing access to the intervention by breaking negotiations

#### Losing access to the community

Throughout my fieldwork, the quantitative portion of my study facilitated access and was readily accepted. This was a double-edged sword, as the findings (and how I chose to share them) started a domino effect resulting in me eventually losing access to the intervention. Near the end of my research, one of my findings was that social capital had increased for the intervention participants, but importantly, that those who participated in the intervention already had higher amounts of social capital to begin with. In the rest of the community, social capital had declined that year. I had initially presented these findings to facilitators and then obtained permission to present these results to participants. After presenting, I asked them what they thought, whether they could think of any explanations, and how we could continue to build on this to improve the intervention. In other words, how could the intervention include people who did not already have high amounts of social capital?

Many of the participants vocally disagreed with the findings. Some asked questions about how the research was done, the scales used, and the overall validity of the results. Others stressed that the intervention had benefitted them individually. I made the mistake of acknowledging that while it benefited some people, some survey respondents indicated feeling excluded by the intervention. I could sense some of the more active participants felt deflated by this information. The facilitators sensed it was a sensitive issue and took the discussion over. Later, my supervisor told me that I had not sugar-coated the findings enough. The COVID-19 pandemic started shortly after this interaction, and I did not have contact with the residents after this point. Looking back, I thought I lost access because sharing my findings meant that I was *no longer participating in their community in a way that was acceptable to them*.

#### Losing access to the facilitators

A few months after I had completed my fieldwork, a facilitator told me that presenting my research had been distressing to participants. From what the facilitator told me, participants had approached them in fear that they might lose funding because of my results. I knew that the facilitators were in a delicate position: They wanted to address issues to improve the intervention, but they also needed to protect participants. In one sense, my work was no longer validating what the facilitators were doing, hence breaking another negotiation.

I regret that I broke the facilitators’ trust after they attended my public Ph.D. examination. One of my examiners had recently left academia and was working for the government, and unfortunately, was also the most critical of the intervention. Afterwards, a facilitator told me that they had understood my need to share findings with other academics to obtain my degree, but that they had not expected a decision-maker to also be present. I had not considered how it would make the facilitators feel, or that they would think it would pose a risk to their livelihoods.

#### Losing access to the organization

I ended up losing access to the intervention when I broke the facilitator's trust. While I had already defended my thesis, there had been a possibility to collect another wave of data once the intervention had been completed, albeit with another student. The facilitators informed me that they had spoken to executives and that the final wave of data collection would no longer be necessary. I had broken the negotiation that the organization could *benefit from my work in a reinforcing* way, leading them to withdraw from future research.

Overall, I gained access to a CBPR at the formal organizational level, starting with a negotiation that I could study the intervention if my findings would benefit them. Looking back, I see that our respective understandings of this negotiation diverged in terms of what *benefit* means. I thought the benefit was to provide research tools and help generate knowledge that would contribute to transformational CBPR, but I also felt that the negotiations relied on my ‘being useful’ to support this [7]. What I failed to recognize was that transforming communities for the better also relied on transforming an organization that was hoping my findings would support its existing agenda and CBPR funding. I ultimately lost access when I broke trust with participants and facilitators, who felt that my findings could have negatively impacted their funding. The risks and costs of continuing to collaborate with researchers in the CBPR process (i.e., the risk of negative findings being public) outweighed any benefits.

## Discussion

While trying to access the intervention, I highlighted negotiations I engaged in across levels: formal organizational with intervention leadership, implementational with facilitators, and community/group level with participants. Along the way, I made multiple negotiations that were reciprocal and embedded within the social networks I developed: I could conduct research if the organization, the facilitators, and the participants benefited from my being there. My interactions within these levels relied on the vertical and horizontal buy-in (and out) from individuals embedded in these levels. An example of vertical buy-in was when the organizational access resulted in access to facilitators, whereas vertical buy-out was when I broke facilitator trust which led to the organization withdrawing. On the other hand, horizontal buy-in and out had more to do with interpersonal relationships: if one participant could vouch for me, their friend might also agree to talk to me. These interactions involved different sources of power and positionality that enabled others to grant and revoke my access to the intervention.

I learned access was constantly negotiated across these boundaries. Access is not static or linear, with varying degrees of access with each person I interacted with. My degree of access depended on my ability to build relationships, which was heavily influenced by who I was, who I knew, how much effort I put into accessing the intervention, and how successful my efforts were. While this is true for most research, I had an added layer of negotiations that a local researcher would not have faced because of my identity as an outsider. I had temporarily convinced the community to let me work with them until I broke trust when I identified and shared issues that made me more of a risk than an asset. The process of negotiating, maintaining, and then losing access helped me understand how interconnected and subjective access is, especially when it comes to developing social capital.

### The dark sides of social capital and CBPR

I have described how negotiating and maintaining access to a CBPR designed to improve social capital in a community relied on individual sources of social capital to take part. This mirrored the somewhat critical findings from my research, which ironically, was one reason I lost access to the intervention.

While social capital researchers have looked at the dark side of social capital (when social relationships that are beneficial to some are detrimental to others) [14, 15], this dark side of building relationships has been less explored from a CBPR perspective. Some researchers have touched on this by highlighting the complexity of building and maintaining trusting relationships in CBPR while also accounting for history and pre-context [9]. Others have looked at the importance of reflecting on social relationships to investigate power and positionality in research that challenges injustice [10]. However, I argue that relationships are almost always considered a positive attribute when it comes to CBPR, overlooking that detrimental social relationships can just as easily arise through the CBPR process.

A key contribution of this work is exploring the connection between social capital and CBPR. Engaging with the idea that there is a dark side to social relationships and the power within them can help dismantle structural issues that are embedded within CBPR. Research has highlighted the power inequalities in social capital interventions [26], and I argue that also a dark side of accessing CBPR because it relies so heavily on social capital. Those who hold grant access also have the power to leverage, contest, withhold, and withdraw that access over time. Those who seek access may have other sources of power that counteract this to some degree, but differentials in power can lead to processes of exclusion.

### Rethinking power in CBPR

A key principle of CBPR is disseminating findings and knowledge gained to all partners while also including all partners in the dissemination process [27]. Researchers have innate power in classifying and constructing frameworks to interpret and understand data [10], and though partners should be included in this process, partners can face an emotional toll in sharing these findings [28]. I found that these two ideas posed an ethical dilemma when partners disagreed with what knowledge was appropriate and whom it could be shared with, particularly when we found that the CBPR was benefiting already privileged people at the expense of others.

Existing guidelines to navigate relationships for CBPR were not fully applicable to my research [27, 29]. CBPR guidelines assume that the researcher holds multiple dimensions of power. First, it assumes that researchers are leading the CBPR process itself (often associated with funding, perceived benefit, and coordinating relationships), but I was a partner collaborating on a community-led project. Second, CBPR assumes researchers hold power related to their identity (e.g., White, cis-gendered, able-bodied, etc.), and that they can ‘empower’ or share power with those from marginalized communities. This assumes that a person in a position to be a researcher would not study a community that holds more power than them. Even though I held some power, I also experienced racism and xenophobia while doing research.

A key issue is that CBPR as a process assumes an ‘ethic of inclusion’ that disrupts white supremacy and racist structures [25]. However, I learned that depending on who funds, leads, and can access CBPR, CBPR can also be used as a tool that upholds dominant power structures and can oppress marginalized peoples. Other CBPR research has found that social systems and structures can even lead participants from racialized backgrounds to uphold these structures during CBPR (e.g., due to internalized racism) [30]. Other researchers have also challenged assumptions of who holds power in CBPR through a reflexive analysis of CBPR in cross-cultural settings [31, 32].

### Moving forward

Questioning assumptions in CBPR through reflexive analysis of the CBPR process before, throughout, and after the project is helpful. I encourage CBPR researchers to consider how social capital plays a role in their CBPR and how this may shift throughout an intervention. This includes looking at community identities, positionality, and the multiplexity of power dynamics that play a role in building mutually trusting relationships required to conduct CBPR. Ultimately, how access is negotiated, how appropriate these negotiations are, and how these negotiations are interpreted will vary from place to place and from person to person. Researchers should continue sharing experiences of how access is negotiated in diverse settings, what sources of capital are needed to engage in these negotiations, and how relevant sources of power are attached to individual and community positionality.
